# Career Satisfaction and Burnout among American Muslim Physicians

**DOI:** 10.1055/s-0043-1770701

**Published:** 2023-07-03

**Authors:** Sondos Al Sad, Aasim I. Padela

**Affiliations:** 1 Initiative on Islam and Medicine, Brookfield, Wisconsin, United States; 2 Department of Emergency Medicine, HUB for Collaborative Medicine, The Medical College of Wisconsin, Milwaukee, Wisconsin, United States

**Keywords:** religiosity, religious accommodations, diversity, inclusion, healthcare workforce

## Abstract

**Background** Career satisfaction and burnout among physicians are important to study because they impact healthcare quality, outcomes, and physicians' well-being. Relationships between religiosity and these constructs are underexplored, and Muslim American physicians are an understudied population.

**Methods** To explore relationships between career satisfaction, burnout, and callousness and Muslim physician characteristics, a questionnaire including measures of religiosity, career satisfaction, burnout, callousness, and sociodemographic characteristics was mailed to a random sample of Islamic Medical Association of North America members. Statistical relationships were explored using chi-squared tests and logistic regression models.

**Results** There were 255 respondents (41% response rate) with a mean age of 52 years. Most (70%) were male, South Asian (70%), and immigrated to the United States as adults (65%). Nearly all (89%) considered Islam the most or very important part of their life, and 85% reported being somewhat or very satisfied with their career. Multivariate models revealed that workplace accommodation of religious identity is the strongest predictor of career satisfaction (odds ratio [OR]: 2.69, *p* = 0.015) and that respondents who considered religious practice to be the most important part of their lives had higher odds of being satisfied with their career (OR: 2.21, *p* = 0.049) and lower odds of burnout (OR: 0.51, *p* = 0.016). Participants who felt that their religion negatively influenced their relationships with colleagues had higher odds of callousness (OR: 2.25, *p* = 0.003).

**Conclusions** For Muslim physicians, holding their religion to be the most important part of their life positively associates with career satisfaction and lower odds of burnout and callousness. Critically, perceptions that one's workplace accommodates a physician's religious identity associate strongly with career satisfaction. In this era of attention to physician well-being, the importance of religiosity and religious identity accommodations to positive career outcomes deserves focused policy attention.

## Introduction

Providers' career satisfaction correlates with healthcare outcomes, patient satisfaction, and providers' retention. 1 2 Career satisfaction is defined in many ways, 3 4 5 6 and is impacted by individual, occupational, and systemic factors. Career satisfaction is defined as the overall contentment with one's choice of occupation and is often used interchangeably with job satisfaction, where workplace conditions and dynamics have a huge influence on that sense of contentment. 7 Career satisfaction is a worker's sense of achievement and success on the job and the collection of feelings and beliefs that people have about their current job. It is perceived to be directly linked to productivity and personal well-being. 6

Career dissatisfaction and burnout are at their peak in our healthcare system during the current pandemic leading to a great loss of practicing clinicians. 8 9 A growing body of research shows that burnout among healthcare providers is highly prevalent. System changes, organizational support, workload, collegiality, and individual physician-specific factors contribute to burnout. 10 In addition to higher morbidity and mortality in patients, physician burnout has been linked to self-reported errors and high turnover. 11 12 Callousness is an advanced stage of burnout 13 in the clinical context, often used as an indicator of malignant burnout and associated with poorer outcomes. 14 15

Whereas 70% of individuals in the United States are religiously affiliated, 16 religiosity's effect on career satisfaction is inadequately studied. 17 18 Studies show that religious beliefs impact work and patient care attitudes, contribute to a sense of personal accomplishment, 19 and build resilience among clinicians. 20 Muslim physicians are underrepresented in research, 21 though they comprise greater than 5% of the US healthcare workforce, 22 and religiosity impacts their professional life in many ways. 23 Moreover, in the past two decades, American Muslim clinicians have experienced more racialization, 24 and discrimination in the workplace 25 26 hence, they may be at higher risk of burnout and scrutiny. 27

To fill in critical gaps in the literature regarding religiosity, religious identity, and how that impacts career satisfaction, burnout, and callousness, we examined predictors of career satisfaction, burnout, and callousness among a national sample of Muslim American physicians.

## Materials and Methods

This study is a secondary data analysis of a national survey of Muslim American physicians carried out by one of the authors (AIP) in 2013. Since study methods are described in detail elsewhere, 28 29 30 only the most critical features are noted below. This study received human subjects research approval from the Institutional Review Board of the Biological Sciences Division at the University of Chicago.

### Participant Recruitment and Data Collection

Since national databases of physicians, like the American Medical Association Masterfile, do not collect data on religious affiliation, we drew upon the membership of the Islamic Medical Association of North America (IMANA; *n* =1968 members in 2013) to draw a national cross-section of Muslim physicians. A random sample of 746 members was selected for receiving a mailed questionnaire. Of these 120 were excluded due to nonworking addresses, decedent status, or those not practicing medicine and no longer identifying as Muslim, leaving us with a sample size of 626 potential respondents. This group received up to three mailed survey questionnaires with escalating fiscal and gift incentives for completion and intermittent postcard and email reminders.

### Survey Instrument and Key Measures

The questionnaire included extant validated measures as well as items created de novo. The questionnaire underwent cognitive pretesting with a group of Muslim clinicians and an expert panel review prior to finalization. Outcome domains were career satisfaction, career burnout, and callousness toward people. The perceived career satisfaction was assessed by adapting an item previously used by Nunez-Smith et al. 31 This item stated “thinking very generally about your satisfaction with your overall career in medicine, would you say that you are currently…” with response categories are appropriate Likert type scale from very dissatisfied to very satisfied. Burnout and callousness were measured by items from West et al's work. 32 The items read “Thinking generally about your overall career in medicine, would you say that currently i) I feel burned out from my work ii) I have become more callous toward people since I took this job.” Each item had a 7-point frequency response scale from never to every day.

The primary predictor domain was physician religiosity measured by different constructs. Religious importance 33 was measured with the question “How important would you say your religion is in your life?” with responses from “not important” to “the most important part of my life.” This question has been used in multiple physician surveys assessing religion-associated variations in physicians' clinical practices. 34 35 Religious practice was measured with five items. The first three assessed the frequency with which participants (a) attended congregational worship (daily to less than once per year), (b) performed Islamic ritual prayers (five times per day to never), and (c) read the Qur'an (daily to never). The fourth item assessed the extent to which the participant keeps the Ramadan fasts (strictly to not at all), while the fifth item assessed adherence to Islamic legal injunctions regarding the consumption of meat (participants reported whether they would eat meat slaughtered according to Islamic law, kosher meat, any meat aside from pork, or did not eat meat). To assess their religious appearance, we asked male respondents whether they wore a beard and female respondents whether they wore a hijab. We also asked about sectarian affiliation within Islam (Sunni, Shi'ite). Other predictor domains were perceived religious discrimination at the workplace, religious accommodation at the workplace, and discrimination-related job turnover. 30

Finally, the questionnaire captured conventional sociodemographic descriptors (gender, age, ethnic/racial background, country of medical school matriculation, and generational status in the United States) and practice-level data (years in medical practice, medical specialty, primary work setting, and the percentage of Muslim patients in each participant's practice).

### Data Analyses

Independent double data entry was performed in Research Electronic Data Capture (REDCap) databases, 36 and cross-compared with original surveys to resolve discrepancies. Where possible, variables were transformed for ease of interpretation in the following ways: (i) dichotomizing agreement and satisfaction scales, (ii) collapsing response categories where responses totaled less than 5% of the sample into adjacent categories, and (iii) dropping the “other” response category if this category held less than 5% of the sample. A religious practice variable was a summed score of the five items noted above. After generating descriptive statistics, we used chi-squared tests and simple logistic regression to test bivariate associations between each predictor and outcome variable. Given the exploratory nature of assessing relationships between our variables of interest, bivariate associations significant at the level of *p* -value less than 0.10 were moved forward into final multivariate logistic regression models for each outcome measure, where the conventional *p* -value less than 0.05 threshold indicated a significant association.

## Results

### Participant Characteristics

We had 255 respondents (41% response rate) with a mean age of 52 years. Most were male (172, 69.9%), of South Asian ethnicity (172, 69.9%), completed medical school abroad (166, 69.3%), and had been in medical practice for over 20 years (137, 57.4%). Most considered Islam as the most or a very important part of their life (226, 89.2%), strictly fasted Ramadan (215, 85%), and most reported praying five times daily (158, 63%). Almost half wore a beard (44.4% of men) or hijab (43.7% of women; see Tables 1 and 2 ).

**Table 1:** Participants characteristics ( *n* = 255)

Characteristic	*n* (%)
Age, *n* = 238 24–39 40–55 56–69 70–84	66 (27.7) 58 (24.4) 76 (31.9) 38 (16)
Gender, *n* = 246 Male Female	172 (69.9) 74 (30.1)
Race/Ethnicity, *n* = 247 South Asian Arab or Middle Eastern White/Caucasian Black/African American	172 (69.6) 54 (21.9) 10 (4.1) 2 (0.8)
Residency status, *n* = 244 Participant immigrated to the United States as an adult Participant immigrated to the United States as a child Participant born in the United States	158 (64.8) 39 (16) 47 (19.3)
Participant completed medical school in the United States, *n* = 243	77 (31.7)
Years of medical practice since completion of medical school, *n* = 239 0–10 11–20 21–30 32–41 42–57	66 (27.6) 36 (15.1) 48 (20.1) 58 (24.3) 31 (13)
Importance of religion in respondent's life, *n* = 254 “The most important part of my life” “Very important in my life” “Fairly important in my life”	136 (53.5) 90 (35.4) 25 (9.8)
Frequency of attendance at congregational worship services, *n* = 251 Daily Less than daily but at least once a week Less than weekly but at least once per month Less than monthly but at least once per year	29 (11.6) 116 (46.2) 47 (18.7) 59 (23.5)
Frequency of prayer, *n* = 251 Five times per day Less than five times a day but at least once per day Less than once per day but at least once per week Rarely or never	158 (63) 65 (25.9) 14 (5.6) 14 (5.6)
Frequency of reading the Quran outside of prayer, *n* = 251 Daily Less than daily but on a weekly basis 2-3 times per month On special occasions Rarely or never	79 (31.5) 50 (19.9) 32 (12.8) 48 (19.1) 42 (16.7)
Keeping Ramadan fast, *n* =253 Strictly Somewhat Not at all	215 (85) 31 (12.3) 7 (2.8)
Dietary practices, *n* = 248 Most religious Very religious Fairly religious Not religious	64 (25.8) 74 (29.8) 96 (38.7) 14 (5.7)
Religious appearance, Keep a beard (male only), *n* = 171 Wear the hijab (female only), *n* = 71	76 (44.4) 31 (43.7)

**Table 2:** Summary of predictor variables, *n* = 255

Variable	Median, mean ± SD
Religious practice	2.0, 2.02 ± 0.47
**	*n* (%)
Religious appearance (hijab/beard)	
No	135 (53.9)
Yes	107 (42.0)
Since completing medical school, how often have you personally experienced discrimination at work because of your religion?	
Never	79 (31.0)
Rarely	112 (43.9)
Sometimes to always	60 (23.5)
Have you personally experienced religious discrimination at your current workplace?	
No	214 (83.9)
Yes	36 (14.1)
Have you ever reported concerns about religious discrimination to an employer or a professional body?	
No	239 (93.7)
Yes	11 (4.3)
My religion negatively influences my relationships with colleagues	
Strongly disagree/disagree	229 (89.8)
Agree/strongly agree	23 (9.0)
My religion positively influences my relationships with colleagues	
Strongly disagree/disagree	83 (32.6)
Agree/strongly agree	163 (63.9)
My religion places me under greater scrutiny than non-Muslim colleagues	
Strongly disagree/disagree	132 (51.8)
Agree/strongly agree	117 (45.9)
I struggle to find time for prayer (salat/namaz) at work	
Strongly disagree/disagree	123 (48.2)
Agree/strongly agree	125 (49.0)
My workplace accommodates my religious identity	
Strongly disagree/disagree	68 (26.7)
Agree/strongly agree	179 (70.2)
Patients have refused my care because of my religious identity	
Strongly disagree/disagree	227 (89.0)
Agree/strongly agree	22 (8.6)
Did Islamic values influence your choice of specialty?	
No	178 (69.8)
Yes	71 (27.8)

Abbreviation: SD, standard deviation.

With respect to career satisfaction, 216 respondents (85%) were somewhat or very satisfied with their overall career (see Table 4 ). In terms of burnout, there was a diversity of experiences, with 59 (23%) respondents reporting feeling burnt out once a week or more, and 48 (19%) never experiencing it (see Table 5 ). Similarly, there was a diversity of experiences with callousness, most (165, 65%) never felt this way, but some (21, 8%) experienced it once a week or more (see Table 6 ).

**Table 3:** Respondent career satisfaction, *n* = 255

Variable	*n* (%)
Thinking very generally about your satisfaction with your overall career in medicine, would you say that you are currently:	
Very/somewhat dissatisfied	38 (14.9)
Somewhat/very satisfied	216 (84.7)
Thinking generally about your overall career in medicine, would you say that you are currently burned out from your work?	
Never	48 (18.8)
A few times a year	78 (30.6)
A few times a month or less	68 (26.7)
Once a week or more	59 (23.1)
Thinking generally about your overall career in medicine, would you say that you are currently more callous towards people since you took your job?	
Never	165 (64.7)
A few times a year	29 (11.4)
A few times a month or less	27 (10.6)
Once a week or more	21 (8.2)

**Table 4:** Bivariate associations between predictor variables and career satisfaction

Variable	Very/somewhat dissatisfied, *n* = 38	Somewhat/very satisfied, *n* = 216	
	Mean ± SD		*p* -Value
Religious practice	1.87 ± 0.54	2.05 ± 0.45	0.028
Age (years)	53.9 ± 15.8	51.7 ± 15.8	0.440
**	*n* (%)		*p* -Value
Religious appearance (hijab/beard)			0.506
No	23 (60.5)	111 (54.7)	
Yes	15 (39.5)	92 (45.3)	
Since completing medical school, how often have you personally experienced discrimination at work because of your religion?			0.852
Never	12 (32.4)	67 (31.5)	
Rarely	15 (40.5)	96 (45.1)	
Sometimes to always	10 (27.0)	50 (23.5)	
Have you personally experienced religious discrimination at your current workplace?			0.742
No	31 (83.8)	182 (85.9)	
Yes	6 (16.2)	30 (14.2)	
My religion negatively influences my relationships with colleagues			1.000
Strongly disagree/disagree	34 (91.9)	194 (90.7)	
Agree/strongly agree	3 (8.1)	20 (9.4)	
My religion positively influences my relationships with colleagues			0.067
Strongly disagree/disagree	17 (47.2)	66 (31.6)	
Agree/strongly agree	19 (52.8)	143 (68.4)	
My religion places me under greater scrutiny than non-Muslim colleagues			0.892
Strongly disagree/disagree	19 (54.3)	113 (53.1)	
Agree/strongly agree	16 (14.7)	100 (47.0)	
I struggle to find time for prayer (salat/namaz) at work			0.979
Strongly disagree/disagree	18 (50.0)	105 (49.8)	
Agree/strongly agree	18 (50.0)	106 (50.2)	
My workplace accommodates my religious identity			0.004
Strongly disagree/disagree	17 (47.2)	51 (24.3)	
Agree/strongly agree	19 (52.8)	159 (75.7)	
Patients have refused my care because of my religious identity			1.000
Strongly disagree/disagree	34 (91.9)	192 (91.0)	
Agree/strongly agree	3 (8.1)	19 (9.0)	
Did Islamic values influence your choice of specialty?			0.568
No	28 (75.7)	150 (71.1)	
Yes	9 (24.3)	61 (28.9)	
Percentage of patients who are Muslim			0.495
< 2%	16 (43.2)	72 (35.1)	
2–4.99%	5 (13.5)	31 (15.1)	
5–14.99%	14 (37.8)	73 (35.6)	
> 15%	2 (5.4)	29 (14.2)	
Community work setting			1.000
Urban	17 (46.0)	94 (46.1)	
Suburban	17 (46.0)	94 (46.1)	
Rural	3 (8.1)	16 (7.8)	
Sex			0.200
Female	8 (21.1)	65 (31.4)	
Male	30 (79.0)	142 (68.6)	
Medical school completed in the United States			0.972
No	26 (68.4)	139 (68.1)	
Yes	12 (31.6)	65 (31.9)	
Ethnicity			0.927
South Asian	23 (76.7)	148 (75.9)	
Arab	7 (23.3)	47 (24.1)	
Years of medical practice			0.908
< 11	8 (22.2)	58 (28.7)	
11–20	5 (13.9)	30 (14.9)	
21–31	8 (22.2)	40 (19.8)	
32–42	9 (25.0)	49 (24.3)	
> 42	6 (16.7)	25 (12.4)	

Abbreviation: SD, standard deviation.

**Table 5:** Bivariate associations between predictor variables and career burnout

Variable	Never, *n* = 48	A few times a year, *n* = 78	A few times a month or less, *n* = 68	Once a week or more, *n* = 59	
	Mean ± SD				*p* -Value
Religious practice	2.15 ± 0.41	2.04 ± 0.47	1.98 ± 0.47	1.94 ± 0.50	0.097
Age (years)	61.6 ± 14.4	56.6 ± 14.5	46.0 ± 13.4	44.8 ± 14.7	<0.001
**	*n* (%)				*p* -Value
Religious Appearance (hijab/beard)					0.866
No	26 (57.8)	39 (52.0)	37 (58.7)	32 (56.1)	
Yes	19 (42.2)	36 (48.0)	26 (41.3)	25 (43.9)	
Since completing medical school, how often have you personally experienced discrimination at work because of your religion?					0.158
Never	18 (37.5)	25 (32.5)	22 (32.4)	13 (23.2)	
Rarely	23 (47.9)	31 (40.3)	34 (50.0)	23 (41.1)	
Sometimes to always	7 (14.6)	21 (27.3)	12 (17.7)	20 (35.7)	
Have you personally experienced religious discrimination at your current workplace?					0.052
No	43 (89.6)	66 (85.7)	62 (91.2)	41 (74.6)	
Yes	5 (10.4)	11 (14.3)	6 (8.8)	14 (25.5)	
My religion negatively influences my relationships with colleagues					0.044
Strongly disagree/disagree	43 (91.5)	76 (97.4)	58 (85.3)	50 (87.7)	
Agree/strongly agree	4 (8.5)	2 (2.6)	10 (14.7)	7 (12.3)	
My religion positively influences my relationships with colleagues					0.175
Strongly disagree/disagree	11 (24.4)	24 (31.2)	22 (33.3)	25 (44.6)	
Agree/strongly agree	34 (75.6)	53 (68.8)	44 (66.7)	31 (55.4)	
My religion places me under greater scrutiny than non-Muslim colleagues					<0.001
Strongly disagree/disagree	27 (57.5)	49 (63.6)	39 (58.2)	16 (28.6)	
Agree/strongly agree	20 (42.6)	28 (36.4)	28 (41.8)	40 (71.4)	
I struggle to find time for prayer (salat/namaz) at work					0.007
Strongly disagree/disagree	30 (65.2)	42 (54.6)	33 (48.5)	18 (32.1)	
Agree/strongly agree	16 (34.8)	35 (45.5)	35 (51.5)	38 (67.9)	
My workplace accommodates my religious identity					0.913
Strongly disagree/disagree	12 (26.1)	23 (30.3)	18 (26.9)	14 (25.0)	
Agree/strongly agree	34 (73.9)	53 (69.7)	49 (73.1)	42 (75.0)	
Patients have refused my care because of my religious identity					0.989
Strongly disagree/disagree	43 (91.5)	71 (92.2)	60 (89.6)	51 (91.1)	
Agree/strongly agree	4 (8.5)	6 (7.8)	7 (10.5)	5 (8.9)	
Did Islamic values influence your choice of specialty?					0.154
No	37 (80.4)	53 (68.8)	50 (76.9)	37 (62.7)	
Yes	9 (19.6)	24 (31.2)	15 (23.1)	22 (37.3)	
Community work setting					0.754
Urban	22 (47.8)	36 (48.0)	24 (38.7)	28 (49.1)	
Suburban	22 (47.8)	31 (41.3)	33 (53.2)	25 (43.9)	
Rural	2 (4.4)	8 (10.7)	5 (8.1)	4 (7.0)	
Sex					0.274
Female	9 (19.6)	23 (30.3)	19 (30.2)	22 (37.3)	
Male	37 (80.4)	53 (69.7)	44 (69.8)	37 (62.7)	
Medical school completed in the United States					0.038
No	39 (84.8)	51 (68.0)	37 (59.7)	37 (63.8)	
Yes	7 (15.2)	24 (32.0)	25 (40.3)	21 (36.2)	
Ethnicity					0.752
South Asian	36 (81.8)	50 (74.6)	44 (75.9)	40 (72.7)	
Arab	8 (18.2)	17 (25.4)	14 (24.1)	15 (27.3)	
Years of medical practice					<0.001
< 11	3 (6.4)	12 (16.4)	22 (36.1)	29 (51.8)	
11–20	7 (14.9)	13 (17.8)	9 (14.8)	6 (10.7)	
21–31	10 (21.3)	13 (17.8)	14 (23.0)	11 (19.6)	
32–42	12 (25.5)	25 (34.3)	14 (23.0)	7 (12.5)	
> 42	15 (31.9)	10 (13.7)	2 (3.3)	3 (5.4)	

Abbreviation: SD, standard deviation.

**Table 6:** Bivariate associations between predictor variables and feelings of callousness

Variable	Never, *n* = 48	A few times a year, *n* = 78	A few times a month or less, *n* = 68	Once a week or more, *n* = 59	
	Mean ± SD				*p* -Value
Religious practice	2.06 ± 0.47	1.90 ± 0.49	2.00 ± 0.41	1.77 ± 0.48	0.034
Age (years)	56.8 ± 13.9	44.7 ± 15.8	34.9 ± 8.9	38.4 ± 11.1	<0.001
**	*n* (%)				*p* -Value
Religious appearance (hijab/beard)					0.374
No	85 (54.5)	16 (57.1)	14 (53.9)	15 (75.0)	
Yes	71 (45.5)	12 (42.9)	12 (46.2)	5 (25.0)	
Since completing medical school, how often have you personally experienced discrimination at work because of your religion?					0.615
Never	54 (32.9)	7 (25.0)	9 (34.6)	6 (28.6)	
Rarely	67 (70.9)	16 (57.1)	13 (50.0)	8 (38.1)	
Sometimes to always	43 (26.2)	5 (17.9)	4 (15.4)	7 (33.3)	
Have you personally experienced religious discrimination at your current workplace?					0.541
No	140 (85.4)	25 (89.3)	23 (88.5)	15 (75.0)	
Yes	24 (14.6)	3 (10.7)	3 (11.5)	5 (25.0)	
My religion negatively influences my relationships with colleagues					0.081
Strongly disagree/disagree	151 (92.1)	27 (96.4)	22 (81.5)	17 (81.0)	
Agree/strongly agree	13 (7.9)	1 (3.6)	5 (18.5)	4 (19.1)	
My religion positively influences my relationships with colleagues					0.871
Strongly disagree/disagree	54 (33.8)	9 (33.3)	7 (26.9)	8 (38.1)	
Agree/strongly agree	106 (66.3)	18 (16.7)	19 (73.1)	13 (61.9)	
My religion places me under greater scrutiny than non-Muslim colleagues					0.028
Strongly disagree/disagree	93 (56.7)	15 (55.6)	13 (48.2)	4 (21.1)	
Agree/strongly agree	71 (43.3)	12 (44.4)	14 (51.9)	15 (79.0)	
I struggle to find time for prayer (salat/namaz) at work					0.000
Strongly disagree/disagree	95 (59.0)	14 (50.0)	6 (22.2)	2 (9.5)	
Agree/strongly agree	66 (41.0)	14 (50.0)	21 (77.8)	19 (90.5)	
My workplace accommodates my religious identity					0.552
Strongly disagree/disagree	41 (25.8)	9 (32.1)	6 (22.2)	8 (38.1)	
Agree/strongly agree	118 (74.2)	19 (67.9)	21 (77.8)	13 (61.9)	
Patients have refused my care because of my religious identity					0.452
Strongly disagree/disagree	145 (90.1)	27 (96.4)	26 (96.3)	18 (85.7)	
Agree/strongly agree	16 (9.9)	1 (3.6)	1 (3.7)	3 (14.3)	
Did Islamic values influence your choice of specialty?					0.322
No	121 (75.2)	22 (75.9)	16 (61.5)	13 (61.9)	
Yes	40 (24.8)	7 (24.1)	10 (38.5)	8 (38.1)	
Community work setting					
Urban	61 (39.4)	19 (65.5)	14 (53.9)	10 (52.6)	0.068
Suburban	82 (52.9)	9 (31.0)	8 (30.8)	8 (42.1)	
Rural	12 (7.7)	1 (3.5)	4 (15.4)	1 (5.3)	
Sex					0.228
Female	40 (25.5)	11 (37.9)	9 (34.6)	9 (42.9)	
Male	117 (74.5)	18 (62.1)	17 (65.4)	12 (57.1)	
Medical school completed in the United States					<0.001
No	118 (75.2)	17 (60.7)	10 (40.0)	9 (42.9)	
Yes	39 (24.8)	11 (39.3)	15 (60.0)	12 (57.1)	
Ethnicity					0.866
South Asian	112 (77.8)	20 (74.1)	18 (78.3)	14 (70.0)	
Arab	32 (22.2)	7 (25.9)	5 (21.7)	6 (30.0)	
Years of medical practice					<0.001
< 11	20 (12.9)	13 (46.4)	18 (75.0)	14 (70.0)	
11–20	21 (13.6)	5 (17.9)	4 (16.7)	4 (20.0)	
21–31	40 (25.8)	3 (10.7)	1 (4.1)	1 (5.0)	
32–42	47 (30.3)	6 (21.4)	1 (4.1)	0 (0.0)	
> 42	27 (17.4)	1 (3.6)	0 (0.0)	1 (5.0)	

Abbreviation: SD, standard deviation.

Concerning the perceived impact of religious identity on relationships with colleagues, 9% of respondents felt that their religious identity negatively influenced relationships with colleagues, while 68% felt it was a positive influence.

### Predictors of Career Satisfaction

The strongest predictor of overall career satisfaction was the belief that one's workplace accommodated their religious identity (odds ratio [OR]: 2.69, *p* <0.015). Respondents who had higher levels of religious practice also had higher odds of career satisfaction (OR: 2.21, *p* < 0.049; (see Table 7 )

**Table 7:** Logistic regression model of physician predictors of career satistfaction, a *n* = 240

Predictor	Odds ratio (95% confidence interval)	*p* -Value
Religious practice	2.21 (1.00, 4.87)	0.049
Workplace accommodates my religious identity	2.69 (1.21, 5.95)	0.015
Religion positively influences relationships	1.08 (0.47, 2.46)	0.853

a Thinking very generally about your satisfaction with your overall career in medicine, rate your current satisfaction.

### Predictors of Burnout and Callousness

Participants who were older (OR: 0.94, *p* < 0.000), US medical graduates (OR: 0.44, *p* < 0.011), and those who considered religious practice to be the most important part of their lives (OR: 0.51, *p* < 0.016) had lower odds of burnout (see Table 8 ).

**Table 8:** Logistic regression model of physician predictors of career burnout, a *n* = 221

Predictor	Odds ratio (95% confidence interval)	*p* -Value
Religious practice	0.51 (0.29, 0.88)	0.016
Age	0.94 (0.92, 0.95)	0.000
Discrimination at the current workplace	1.65 (0.75, 3.64)	0.208
My religion negatively influences relationships with colleagues	1.25 (0.48, 3.22)	0.637
My religion places me under greater scrutiny	1.50 (0.85, 2.65)	0.155
Struggle to find time for prayer	1.19 (0.71, 2.00)	0.506
Completed medical school in the United States	0.44 (0.23, 0.82)	0.011

a Thinking generally about your overall career in medicine, how often would you say you feel burned out from your work?

With respect to callousness, respondents who considered religious practice to be the most important part of their lives (OR: 0.42, *p* < 0.024) worked in suburban settings compared with those who worked in urban settings (OR: 0.42, *p* <0.017) and older participants (OR: 0.91, *p* < 0.001) had lower odds of callousness. While those whose religion negatively influenced their relationships with colleagues had greater odds to experience callousness (OR: 2.25, *p* <0.003). Of marginal statistical significance, those who reported struggling to find time for prayer were at higher odds of callousness than those who did not (OR: 1.45, *p* < 0.066; see Table 9 ).

**Table 9:** Logistic regression model of physician predictors on feelings of callousness, a *n* = 211

Predictor	Odds ratio (95% confidence interval)	*p* -Value
Religious practice	0.42 (0.20, 0.89)	0.024
Age	0.91 (0.89, 0.94)	<0.001
My religion negatively influences relationships with colleagues	2.25 (1.31, 3.86)	0.003
My religion places me under greater scrutiny	1.17 (0.76, 1.80)	0.485
Struggle to find time for prayer	1.45 (0.98, 2.16)	0.066
Community setting	1.61 (0.86, 3.03)	0.139
Urban	REF	REF
Suburban	0.42 (0.21, 0.86)	0.017
Rural	1.02 (0.29, 3.62)	0.979
Completed medical school in the United States	0.61 (0.29, 1.31)	0.207

a Thinking generally about your overall career in medicine, would you say that you are currently more callous towards people since you took your job?

## Discussion

Our national survey shows that religiosity boosts career satisfaction and guards against career burnout and callousness that echo the extant literature as well. 37 38 39 It is no surprising that workplace accommodation of physicians' religious identity contributed to the highest odds of career satisfaction for American Muslim physicians.

The importance of religiosity to healthcare workers is not alike for all religious backgrounds. Muslim physicians were found to value religiosity most and deem it as foundational to their well-being compared to physicians from other religious affiliations. 40 This inherent individual construct comes at no cost to the healthcare system, and while these findings are intuitive to interpret, it appears challenging to implement in healthcare systems. 41

Providing the opportunity to Muslim physicians to practice their faith fosters career satisfaction and well-being. Our participants' struggle to find time for prayers predicted career burnout as well as career satisfaction that reflects the interconnectedness of both burnout and satisfaction even if they may not fall on a linear spectrum. 42

Younger age with fewer years in clinical practice significantly predicted career burnout among Muslim physicians. Physician's age was not a consistent predictor of burnout in the literature but when it was associated, younger age and fewer years in clinical practice were likely to coincide with career burnout. 43 44 This could be a result of the increased administrative load, being on the learning curve to acquire expertise and accomplish tasks, and longer working hours negatively impacting work–life balance. 45 For Muslim physicians, it can be rather confounded by inadequate mentorship, sponsorship, or equitable support from workplaces compared to their physician counterparts.

Those who graduated from US medical schools were less likely to experience career burnout, which may be attributed to their acquaintance with the healthcare system, mentorship networking, and English proficiency with no accent. However, our finding is not consistent with the literature reviewed 46 international medical graduates' experiences may need further research to unpack. 47 It is worth reading about how Muslim international medical graduates may share their equivocal experiences in cultural exchange.

Workplace camaraderie and collegiality can manifest in many ways; Muslim physicians who reported religiosity positively influenced their work relationships were more satisfied in their careers compared to those who reported that their religiosity places them under greater scrutiny. Those who reported being under greater scrutiny are more likely to experience career burnout, and those who reported that their religiosity negatively influenced their work relationship were more prone to callousness. Active involvement and inclusion of Muslim clinicians in organizational cultures, 48 providing professional growth and development resources, 49 and examining the diversity and inclusivity of performance evaluation strategies can improve their workplace experiences. 50

Majoritarianism prevails in the healthcare ecosystem as it does in policymaking, which is palpable in the underrepresentation of Muslim healthcare workers and what may exclusively matter to them. One of the Healthy People 2030 goals is to strengthen the workforce by promoting health and well-being as a high-priority public health issue that does not yet have evidence-based interventions developed to address it. 51 Our study findings suggest that workplace accommodations of religious practice would boost Muslim clinicians' well-being and career satisfaction. 52 53 Given the biases and discrimination pressures that Muslims now face in the United States, focusing policy attention on accommodating Muslim physicians' religiosity is important to enhance their career satisfaction and counteract burnout.

We have developed cutting-edge technology in biomedicine but have had less success in addressing our spirituality. Looking at the issue of physician burnout through the prism of rational problem-solving will highlight that the problem needs to be tackled by incorporating preventative strategies such as mentorship, inclusion, and embracing diversity in addition to developing curative strategies. More importantly, the interventions need to be introduced early in careers rather than at the breaking point. 54

## Limitations

As with any survey-based research, our findings should be interpreted in light of the limitations of the measures used. In particular, the measure of practice-based religiosity represented a summed score of different Islamic practices with variable significance. We acknowledge other practices could have been measured, yet our measure mirrored existing practice-based religiosity measures used in other population research. Further research should explore how various practices associate with career satisfaction, callousness, and burnout.

## Implications

Our national survey of Muslim physicians shows that religiosity positively influences career satisfaction, and potentially reduces career burnout. Workplace accommodations of their religiosity highly predict career satisfaction, whereas being exposed to scrutiny at work and being alienated due to their religiosity predispose them to career burnout and callousness. The Healthcare system should aim toward inclusive workplaces through accommodating religious identity, counteracting occupational scrutiny, and offering cultural training. Although this cross-sectional study cannot be used to make definitive causal inferences, physicians who consider themselves religious are more likely to embrace their careers when workplaces cater for them and are rooted in an inclusive environment that would shield them from burnout and callousness.
